# Nocturnal emergence facilitated by thermally‐induced hatching in the Chinese softshell turtle, *Pelodiscus sinensis*

**DOI:** 10.1002/ece3.9922

**Published:** 2023-03-23

**Authors:** Qingjun Zhu, Liu Lin, Fei Kong, Ting Zhang, Hai‐Tao Shi

**Affiliations:** ^1^ Ministry of Education Key Laboratory for Ecology of Tropical Islands, College of Life Sciences Hainan Normal University Haikou China; ^2^ Shaanxi Provincial Institute of Zoology Xian China

**Keywords:** antipredatory behavior, emergence, freshwater turtle, nest temperature, shallow nest, synchronous hatching

## Abstract

The coincidence of hatching and emergence events with favorable conditions is crucial for turtle survival. Nocturnal emergence has been widely documented across marine and freshwater turtles, and has long been suggested as an adaptive behavior that reduces risks of heat stress and predation. To our knowledge, however, studies related to nocturnal emergence have mainly focused on the post‐hatching behaviors of turtles, and very few experimental studies have been performed to investigate the effects of hatching time on the distribution of emergence times over the course of a day. Here, we visually monitored the activity of the Chinese softshell turtle (*Pelodiscus sinensis*)—a shallow‐nesting freshwater turtle—from hatching to emergence. Our study provides evidence for the novel finding that (i) the timing of synchronous hatching events in *P. sinensis* coincides with the time of day when nest temperatures decrease, (ii) the synchrony between hatching and emergence may further facilitate their nocturnal emergence, and (iii) synchronous behaviors of hatchlings in the nest may be effective in reducing the risk of hatchling predation, and predation is more likely to occur in the asynchronous hatching groups. This study suggests that the hatching of shallow‐nesting *P. sinensis* in response to temperature changes in the nest might be an adaptive nocturnal emergence strategy.

## 1 INTRODUCTION

Embryos of many reptile species benefit from altering the timing of their hatching in response to optimal ambient conditions, which is well‐known as environmentally cued hatching (ECH; Colbert et al., 2010; Doody, 2011; McGlashan et al., 2012; Spencer & Janzen, 2011; Warkentin, 2011). In turtles, evidence is also accumulating for the widespread occurrence of ECH (Doody, 2011). A variety of environmental cues could act as triggers, including the flooding of terrestrial nests (via a hypoxic cue; Doody et al., 2001), mechanical vibrations (Doody et al., 2012), temperature changes (Iverson, 2010), predation (Doody et al., 2012) in freshwater turtles, and possibly vocalizations (Ferrara et al., 2014) in marine turtles. Turtles exhibit synchronous hatching, early hatching, and delayed hatching by responding to these cues (Doody et al., 2012). This plasticity in hatching timing can significantly affect the chance of survival of turtle hatchlings in the short‐term, by reducing predation pressure on individuals and reducing the time and individual energy requirements to dig out from the nest and emerge (Carr & Hirth, 1961; Rusli et al., 2016; Santos et al., 2016), or in the long‐term, by conferring the ability to exploit suitable environmental conditions and enhancing the size and performance of individuals (Arnold & Wassersug, 1978; Spencer & Janzen, 2011).

Even though turtle embryos may hatch in response to optimal conditions, the post‐hatching emergence process is still the most critical period, as hatchlings are extremely vulnerable to predation and unfavorable external conditions (Janzen et al., 2000; Santos et al., 2016). In turtles without parental assistance, post‐hatchlings will need to determine the optimal time for emergence on their own. A well‐documented, efficient approach that matches hatchling emergence with favorable conditions is the adaptation of responding to environmental changes during the process of nest escape (Drake & Spotila, 2002; Glen et al., 2006; Mrosovsky, 1968). For example, marine turtle hatchlings are widely known to emerge synchronously from their nests at night (Bustard, 1967; Glen et al., 2006; Mrosovsky, 1968), as it reduces their risks of dehydration, overheating and predation (Drake & Spotila, 2002; Santos et al., 2016). The selection of emergence timing occurs during the process of digging to the surface when hatchlings wait at a depth that allows them to perceive a decline in temperature associated with night‐time. In this process, thermal cues have been proposed as the main determinants of nocturnal emergence in marine turtles (Drake & Spotila, 2002; Glen et al., 2006; Gyuris, 1993; Moran et al., 1999; Mrosovsky, 1968). Hatchlings of some freshwater turtle species also emerge primarily at night, and temperature is likewise thought to provide the thermal cue for nocturnal emergence (Doody et al., 2001; Plummer, 2007).

The timing of emergence is a life‐history trait influenced by the trade‐off between the risks and benefits of emergence. Natural selection favors hatchlings emerging immediately after hatching or awaiting an environmental cue (e.g., several days from hatching to nest emergence in marine turtles, Carr & Hirth, 1961; delayed emergence of several months in the nest in some freshwater turtles, Gibbons & Nelson, 1978; Lovich et al., 2014; Riley et al., 2014), which indicates a high probability of ensuing favorable conditions (Gibbons, 2013). However, to date, studies related to environmentally cued emergence have mainly focused on the post‐hatching behaviors of turtles (Doody et al., 2001; Drake & Spotila, 2002; Glen et al., 2006; Mrosovsky, 1968; Plummer, 2007), and very few experimental studies have been done to investigate the effects of hatching time on the distribution of emergence times over the course of a day. Hence, our understanding of the process from hatching to emergence in turtles is far from comprehensive (Nishizawa et al., 2021).

In a prior study, we discovered that a shallow‐nesting (vertical nest depth of approximately 10.5 cm) freshwater turtle species, the Chinese softshell turtle (*Pelodiscus sinensis*), exhibits nocturnal emergence in most cases (Zhu et al., 2021) and that their hatchlings do not linger in the nest for more than a day (Qingjun Zhu, personal observation). Considering these observations, the present study aimed to determine the following three exploratory goals: (i) whether daily temperature variations in the shallow nests cued hatching in *P. sinensis*. We hypothesized that shallow nests mirror external temperatures and provide a hatching cue (Doody et al., 2001; Radder & Shine, 2006); (ii) whether the nocturnal emergence of hatchlings is further facilitated by the embryo's selection of hatching timing. We hypothesized that emergence is triggered immediately after hatching (Gibbons, 2013; McGlashan et al., 2018), and the synchrony between hatching and emergence in shallow nests allows the timing of hatching to further influence the timing of emergence (Radder & Shine, 2006); and (iii) the correlations between synchronous behaviors in the nest and the presence of predators.

## 2 MATERIALS AND METHODS

### 2.1 Experimental design

We collected 12 adult female *P. sinensis* (700–1500 g) from the Yellow River in northwestern China (34.95°N, 110.25°E) in May 2021, and kept them in a preset enclosure to lay their eggs. The enclosure (20 m length, 10 m width, 1.2 m height) was set up in their natural habitat (Zhu et al., 2021) and was made of 5 × 5 cm wire mesh (a lattice large enough to allow hatchling dispersal), including a water area of about 10 × 5 m and a land area of 10 × 15 m. We monitored the nest sites through four solar‐powered infrared camera systems (EXF‐HSD, Enxun Electronic Technology, Shenzhen, China) hung on 2.5 m‐high poles around the enclosure.

Shortly after determining where females laid their eggs, we excavated the nest and counted the number of eggs in each nest. To measure the nest and surface temperatures during the incubation period, two temperature probes (GSP‐6, Jingchuang Electronics, Jiangsu, China) were placed at the top and bottom ends of a vertical wooden stake with a length equivalent to the depth of the nest, and then, we reburied the eggs (replacing them in approximately their original positions) within 24 h while placing the stake vertically in the nest. The probes were set to record synchronously every 10 min to an accuracy of 0.1°C. We used only one temperature recording station because *P. sinensis* lay their eggs on flat ground lacking vegetation cover (Zhu et al., 2021), so each nest in the enclosure experiences the same sun exposure, and thermal differences between nests are negligible (Qingjun Zhu, unpublished data).

**FIGURE 1:**
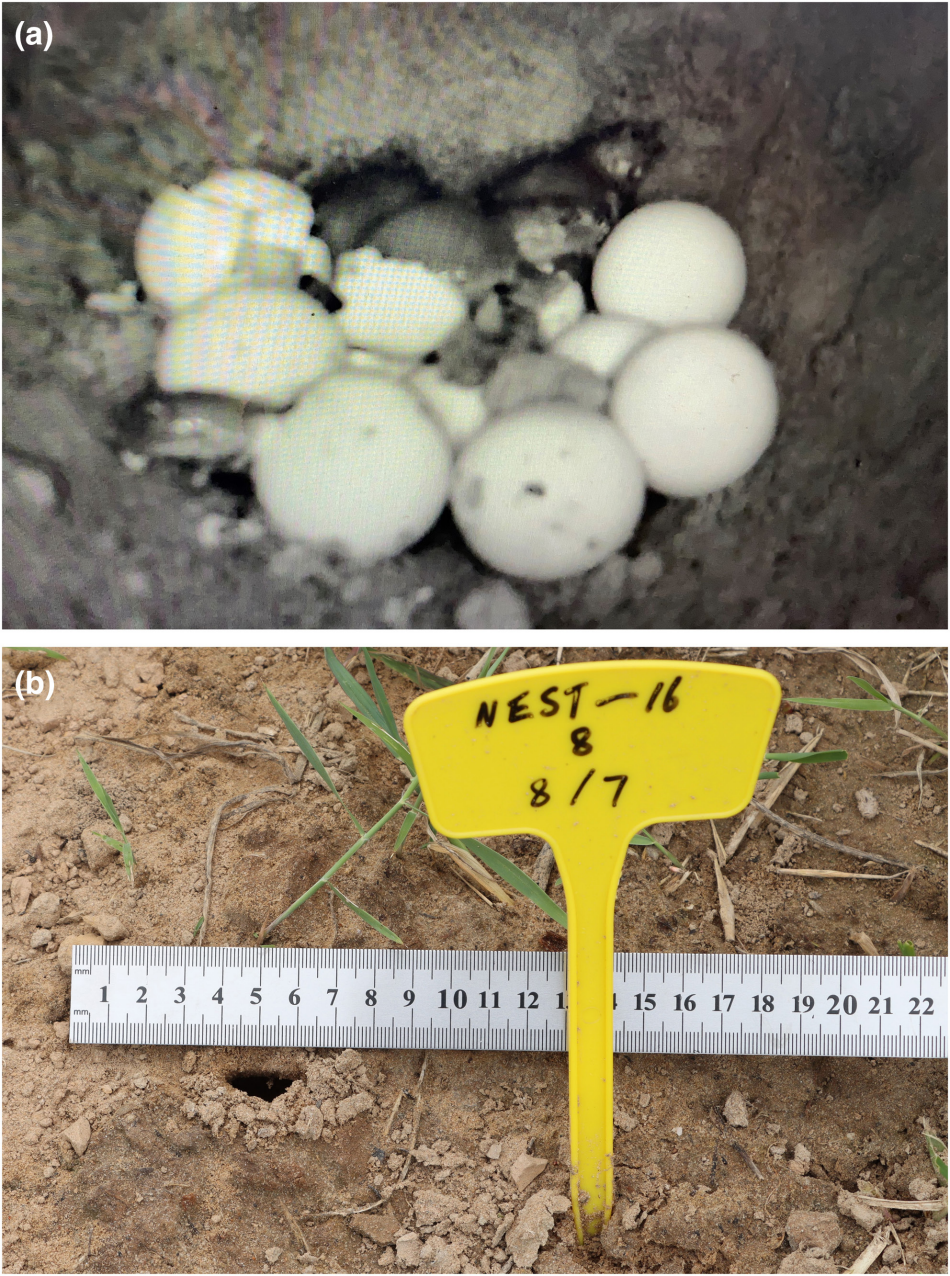
Hatching and emergence patterns of *Pelodiscus sinensis* hatchlings. Synchronous hatching within a nest (a); all the hatchlings from a single nest emerged to the surface from a common passage and opening (b).

One week before the estimated hatching day (mean incubation period = 54.8 days, Zhu et al., 2021), we excavated the nests again and installed a small infrared camera device (EXF‐P3‐4G, Enxun Electronic Technology) in each nest to monitor the hatching process of the embryos. The nests were refilled with sandy soil after equipment installation, and a thin ceramic tile (15 cm length, 10 cm width) was placed across the center of the nest (above the eggs) to prevent the soil from covering the camera equipment (Figure 1). These cameras were connected to solar panels on the ground by wires. The hatching process was recorded as a video and transmitted to a receiver in the laboratory 5 km away through a 4G cellular signal. Although the hatchlings were unable to reach the surface vertically due to obstruction by the tile, all of them emerged from the nest by digging through the soil at the edges of the tile. The subsequent emergence events were captured by the camera outside the nest (EXF‐HSD) and recorded in the SD card. Two days after observing the hatching event, we replaced the SD card of four cameras outside the nests to check the emergence behaviors of the hatchlings. All of the captured individuals were released into the wild after the study.

### 2.2 Statistical analysis

We recorded each hatching, emergence, and predation event by reviewing the video daily, together with the time, nest, and surface temperature of each event. For the temperature data, the reading of the most recent temperature record at the time of an event was used. The rate of change in nest temperatures (°C/h) was obtained by subtracting each recorded temperature from that recorded 1 h prior. Nests hatching in rain were excluded from this study, because rainfall may affect nest temperature (Lolavar & Wyneken, 2015), and hypoxia caused by heavy rain may induce hatching (Doody, 2011; Warkentin, 2011).

To test the correlations between nest temperatures and hatching events, we calculated the average sand temperatures and nest cooling rates every hour using the data recorded for all hatching days (except for missing temperature data on July 24 because of thermometer replacement). The relationship of the number of hatching events with the temperature and nest cooling rate was modeled using a generalized linear mixed model (GLMM) assuming a Poisson distribution. We considered differences between nests as random effects. Because each rate of temperature change varied in frequency over the course of a day, to clearly determine the effect of various temperature changes on hatching we used the method of Glen et al. (2006) to define a relative hatching index (RHI), which indicates the relative proportion of hatching that occurred at each nest‐temperature decrement (increment). For example, 47.1% of eggs in nests hatched when the rate of cooling was 0.3–0.4°C/h, which accounted for 24.8% of the time. Therefore, the RHI was 1.9 (47.1/24.8). The larger the RHI, the greater the effect of the rate of temperature change on hatching events.

To test hatching synchrony and synchrony between hatching and emergence, we counted the number of hatched eggs left in all corresponding nests every minute after the first egg hatched in each nest. The number of hatched eggs in each nest was analyzed as a function of time (every minute after the first egg hatched) using a generalized linear model with a Poisson distribution. We calculated the time from the first embryo pipped to the first hatchling emerged, and the synchrony between hatching and emergence was assessed using these time intervals in each nest.

Predation risk was assessed in the two groups comprising synchronous hatching and asynchronous hatching (predation events that occurred outside the nest were not counted). We considered the synchronously hatched hatchlings as the first group and all asynchronously hatched hatchlings as the second group. The relationship of the number of predation events with the population size of the group and the order of the groups was modeled using a GLMM assuming a negative binomial distribution. All analyses were performed using SPSS 26.0.

## 3 RESULTS

### 3.1 Hatching timing and nest temperature related to hatching

**FIGURE 2:**
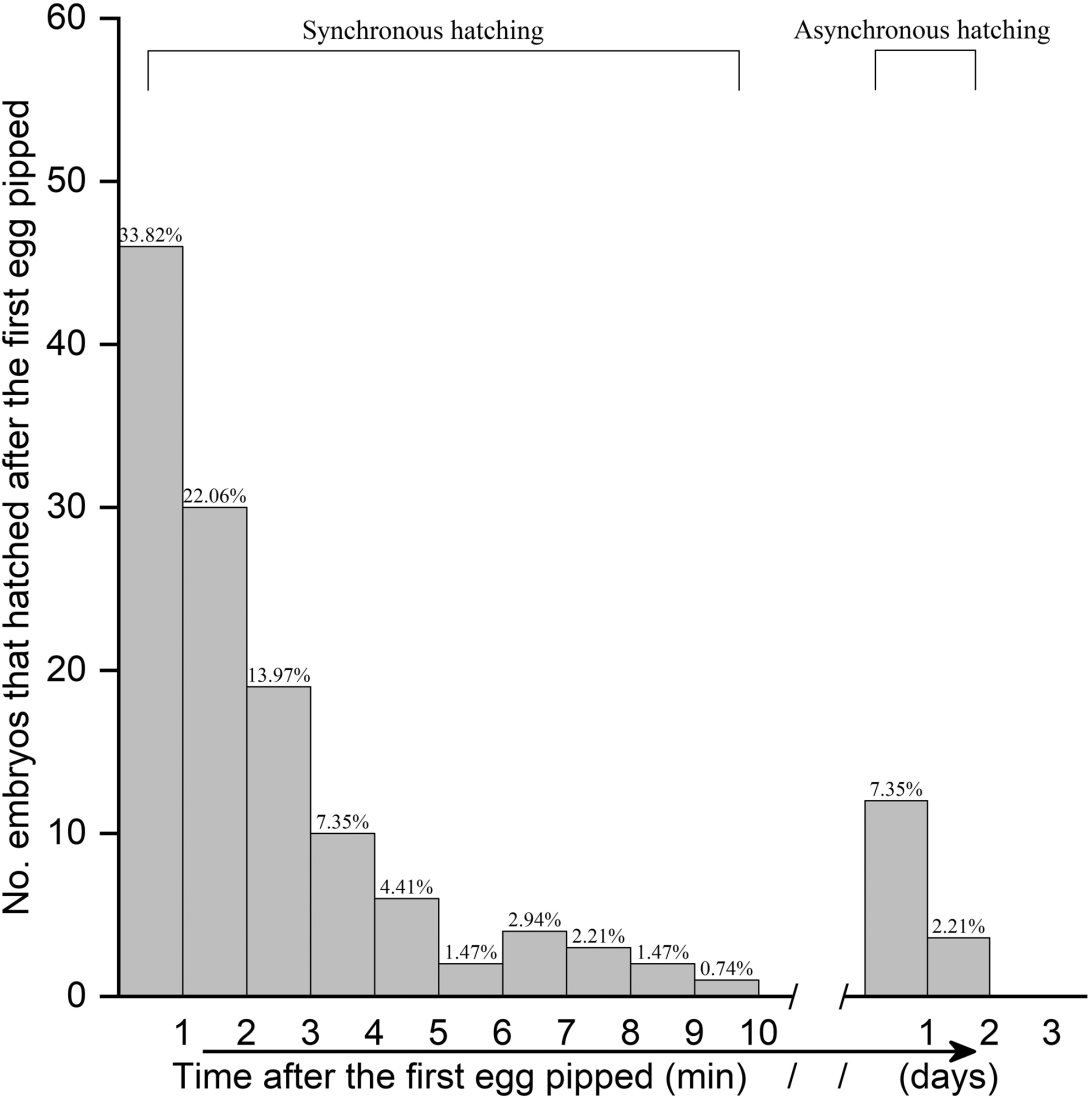
Prediction of synchronous hatching in *Pelodiscus sinensis*. We calculated the total number of hatched eggs left in all corresponding 17 nests every minute after the first egg hatched in each nest. Generally, when an egg in a nest first hatched, 90.44% of the remaining eggs in all nests pipped successively within 10 min. During that time, the number of embryos that hatched decreased accordingly as time passed (*X* ^*2*^ = 18.189, *p* < .001).

In total, 153 hatchlings hatched from 197 eggs in the 17 nests that were constructed by the 12 females (some females deposited multiple clutches of eggs), after an incubation period ranging from 52 to 65 days. In general, when an egg in a nest hatched first, the remaining eggs in each nest pipped successively within 10 min (Figure 1a). During that time, the number of embryos that hatched decreased accordingly as time passed (*X* ^*2*^ = 18.189, *p* < .001), indicating a synchronous hatching of eggs (Figure 2). In a few exceptional cases (9.56%), 13 eggs in five nests hatched 1–2 days after the first egg in the corresponding nest pipped (Figure 2).

**FIGURE 3:**
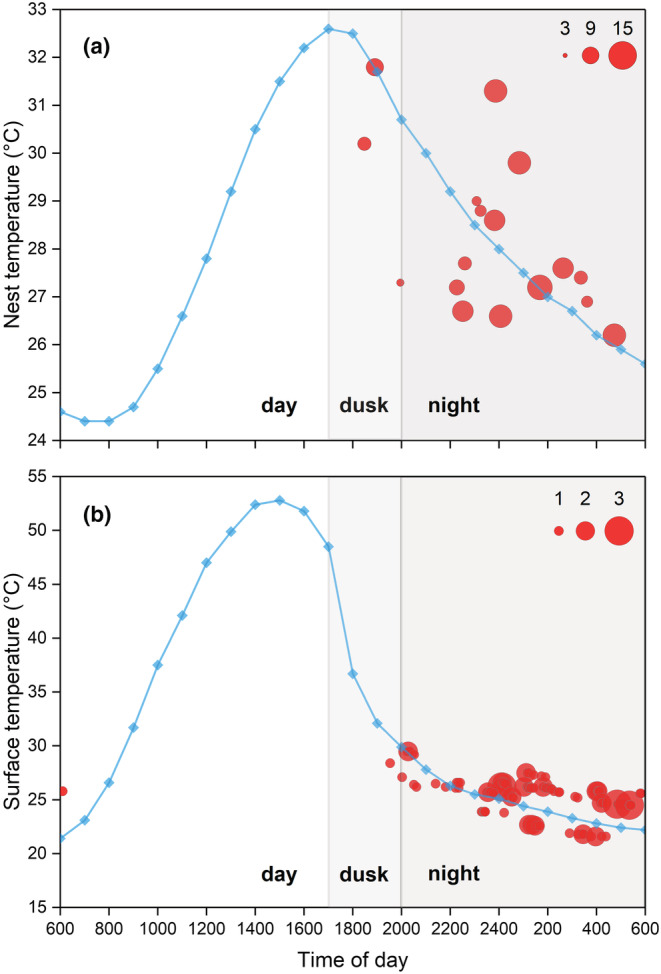
Temporal distributions of hatching and emergence events. The horizontal coordinates of each bubble reflect the timing of the synchronous hatching event in each nest (a) and the time of the hatchling emergence (b), respectively; the vertical coordinates of each bubble reflected the nest temperature when hatching occurred (a) and the surface temperature when hatchlings emerged (b), respectively. The bubble size represents the number of individuals that hatched synchronously (a) and the number of hatchlings that emerged simultaneously (b). The blue line indicates nest (a) and surface (b) temperatures on a representative hatching day (29 July 2021).

**FIGURE 4:**
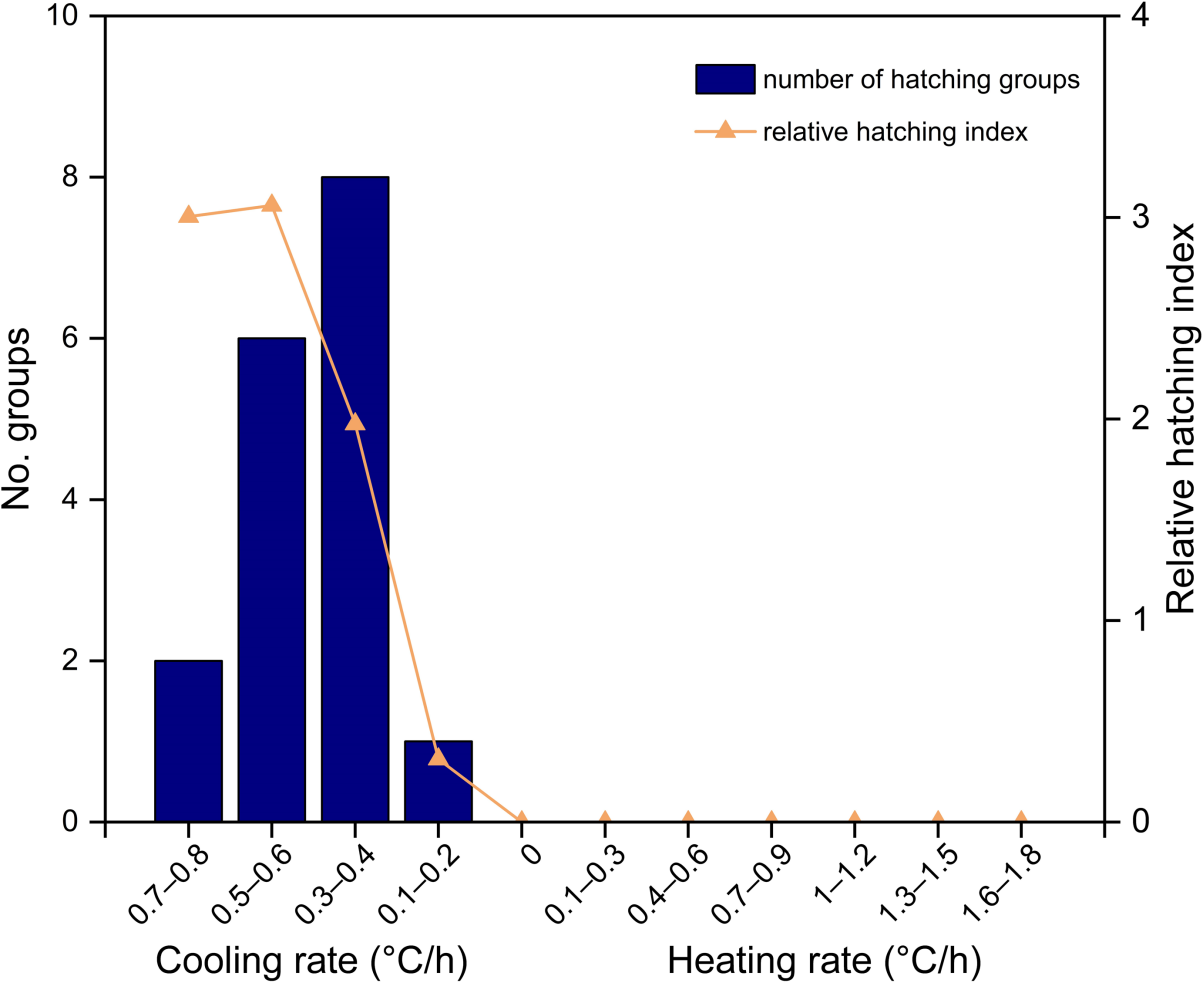
The number of hatching groups and the relative hatching index (RHI) under different rates of nest‐temperature change. The RHI was greater during the stage when nest temperatures decreased rapidly (cooling rate > 0.5°C/h) than when nest temperatures decreased gradually, indicating that hatching was more likely to occur during the period of rapidly decreasing nest temperatures. (*Note*: the cooling rate of nest temperatures ranged from 0.1 to 1°C/h, but no embryos hatched when the cooling rate was 0.9–1°C/h. The rare occurrence (1.94%) of this cooling rate could explain such a situation).

Synchronous hatching events occurred at dusk (17:00–20:00; 18% of the total nests) or at night (20:00–6:00; 82% of the total nests) when the nest temperature was decreasing (Figure 3a). The GLMM analysis indicated that there was a significant effect of the rate of nest cooling on the occurrence of synchronous hatching events (*F*_1,405_ = 5.463, *t* = −2.337, *p* = .020), instead of the nest temperature (*F*_1,405_ = .170, *t* = −0.412, *p* = .680). The eggs in most nests hatched when the rate of nest cooling was 0.3–0.4°C/h (Figure 4). However, the RHI was greater during the stage when nest temperatures decreased rapidly (rate of cooling > 0.5°C/h) than when nest temperatures decreased gradually (Figure 4), indicating that pipping was more likely to occur during the period of rapidly decreasing nest temperatures.

### 3.2 Synchrony between hatching and emergence and timing of emergence

No delayed emergence was found in hatchlings, with the time interval between the hatching of the first egg and the emergence of the first hatchling in each nest being 67 ± 16 min (mean ± SD, range = 22–88), as shown in Table 1. Subsequently, after the time interval between hatching and nest emergence, almost all hatchlings emerged at night and in the early morning, when the surface temperatures were significantly lower than those during the day (Figure 3b). The hatchlings in a single nest emerged alone, with a median time between emerging individuals of 1.8 min, through the only opening of each nest before crawling singly toward the water source (Figure 1b).

**TABLE 1:** Results of the time interval between hatching and emergence and predation events that occurred in synchronous and asynchronous hatching groups of *Pelodiscus sinensis*.

Nest	Date hatched	Hatching timing	No. hatchlings	Time from the first embryo pipped to the first hatchling emerged (min)	No. hatchlings/no. predation events in synchronous hatching group	No. hatchlings/no. predation events in asynchronous hatching group
1	13 July	1:40	13	73	13/0	0/0
2	14 July	23:51	12	75	7/0	5/2
3	19 July	18:28	7	64	7/1	0/0
4	23 July	0:50	12	59	12/1	0/0
5	24 July	0:04	12	68	12/0	0/0
6	25 July	23:05	5	79	5/0	0/0
7	29 July	18:54	9	80	7/0	2/2
8	8 August	3:21	7	49	7/0	0/0
9	19 August	22:35	7	88	7/0	0/0
10	15 August	22:16	8	61	8/1	0/0
11	20 August	23:11	6	53	6/1	0/0
12	17 August	19:57	4	86	4/0	0/0
13	19 August	2:38	11	80	11/0	0/0
14	20 August	4:43	12	22	10/0	2/0
15	20 August	23:49	11	72	10/0	1/1
16	17 August	22:30	11	59	8/0	3/1
17	21 August	3:36	6	74	6/0	0/0

### 3.3 Predation

Among the 153 hatchlings, eight (5.23%) were surrounded and preyed upon by large numbers of ants inside the nest. Two (1.31%) were preyed on by a mouse and an Asiatic toad (*Bufo gargarizans*) after emergence, respectively. The large colony of ants gathered in the nests approximately 2 h after the first egg had hatched, resulting in higher predation risks in nests with asynchronous hatching than in those with synchronous hatching (*F*_1,19_ = 5.642, *t* = −2.375, *p* = .028), with the population size of the group having no effect (*F*_1,19_ = 1.107, *t* = 1.035, *p* = .322). In addition to hatchlings, these ants fed on the shell or remaining tissues after hatchlings had emerged.

## 4 DISCUSSION

Thermal cues that trigger nocturnal emergence in turtles are primarily assessed in terms of the post‐hatching behavior (Doody et al., 2001; Drake & Spotila, 2002; Glen et al., 2006; Mrosovsky, 1968; Plummer, 2007). Our study provides the first evidence for the novel finding that the timing of hatching coincides with the time of day when nest temperatures decrease (Figure 2), and the synchrony between hatching and emergence may further facilitate the nocturnal emergence in a freshwater turtle species. Our results also indicate that synchronous behaviors of hatchlings in the nest may be effective in reducing the risk of hatchling predation.

We hypothesized that full‐term *P. sinensis* embryos within eggs could determine the timing of their hatching in response to environmental cues that can be detected within the subterranean nest, as has been previously suggested (Radder & Shine, 2006). Surprisingly, we found that the nest temperature of *P. sinensis* displayed pronounced diel variation, with an appreciable decline from dusk, and that the hatching of the eggs in all nests occurred when nest temperatures decreased (Figure 3a). At first sight, the hatching of *P. sinensis* mainly occurred when the rate of nest cooling was moderate (e.g., 0.3–0.4°C/h; Figure 4). However, this was the primary cooling rate of the nests. Considering this, our intention with the RHI was to determine the effect of various temperature changes on hatching. Intriguingly, the index was greatest at larger nest cooling rates, indicating that hatching in *P. sinensis* was more likely a response to a rapid decrease in nest temperature (Figure 4). Our results reject the possibility that the embryonic response to absolute nest temperatures promotes the nocturnal emergence of hatchlings. However, other environmental cues that might establish a diel cycle cannot be excluded by the results of this study, such as external sound stimuli (Nishizawa et al., 2021; Rusli et al., 2016). To demonstrate a decreasing temperature mechanism more directly, the environment of eggs could be manipulated, for example by using a standardized environment with constant sound, to investigate how embryos respond to changing temperatures.

Additionally, our hypothesis that decreasing temperature triggers hatching and promotes the nocturnal emergence of *P. sinensis* was also based on the synchrony between hatching and the emergence of hatchlings in all nests. Turtle hatchlings benefit from altering their timing of emergence by emerging immediately after hatching or awaiting an environmental cue (some degree of delayed emergence, both short‐term and long‐term; Gibbons & Nelson, 1978; Lovich et al., 2014; Plummer, 2007; Riley et al., 2014) that indicates an optimal condition. The short interval between hatching and emergence in *P. sinensis* indicates that immediate emergence after hatching is the overwhelmingly dominant strategy in this species. However, a potential error source of our experiment is that a section of the nest (above the eggs) was hollowed out, which could have resulted in a shorter time between hatching and emergence and lead to discrepancies between our findings and those of Plummer (2007), who found that smooth softshell turtle (*Apalone mutica*) hatchlings congregated just below the surface to wait for emergence (maybe a short‐term delayed emergence described in his study).

The nature of the synchronous behavior between hatching and emergence requires further investigation. In addition to possible active efforts to promote nocturnal emergence, we hypothesized that pressure from predation may be a potential passive driver. This was supported by the fact that a large colony of ants (the primary predator in this study) gathered in the nests approximately 2 h after the first egg had hatched. Because hatching‐related odors may attract predators (Doody, 2011), hatchlings are most likely more vulnerable to predation than embryos in eggshells, which would pose a survival problem if they hatched during the day and waited for nocturnal emergence. Hence, the synchrony between hatching and emergence may be vital for *P. sinensis* hatchlings, as it is conducive not only to facilitating nocturnal emergence but also to reducing predation risk.

Meanwhile, reducing pressure from predation has been commonly used to explain synchronous behavior in animals, and is widely considered to be one of the main drivers in the evolution of aggregation behavior (Santos et al., 2016). We found that, like many freshwater turtle species, a synchronous hatching mechanism is also present in *P. sinensis*. Synchronous hatching could reduce predation risk through dilution or coordinate departure of hatchlings from the nest, because chemical cues released during hatching could attract predators to the remaining eggs or hatchlings (Carr & Hirth, 1961; Doody et al., 2012; Spencer et al., 2001; Vitt, 1991). Consistent with this idea, the predation risks in nests with asynchronous hatching were higher than those with synchronous hatching in this study.

Nevertheless, our results did not support the hypothesis that synchronous hatching may facilitate synchronous emergence from the nest, as has been previously suggested (Colbert et al., 2010; Doody, 2011; Spencer, 2012). Most *P. sinensis* hatchlings did not emerge in a group or several small groups but crawled toward the water source individually (Figure 2b), a phenomenon that has also been observed in other freshwater turtles (Doody et al., 2001; Plummer, 2007). Synchronous emergence may decrease the chance of predation through the per capita dilution of individual predation risk by swamping predators (Santos et al., 2016; Tucker et al., 2008). By contrast, predation in *P. sinensis* occurred primarily within the nest. The lower risk of predation encountered by the nocturnally emerged hatchlings and the weakness of the swamping effect owing to the relatively small population size (average of nine hatchlings per nest) probably resulted in the synchrony of emergence not being a primary selective agent in this species. Instead, hatchlings may avoid predators by emerging from the nest immediately after hatching and entering the water as soon as possible. However, synchronously hatched individuals may still have synergistic or facilitative effects on one another, involving jointly digging a passage out from the nest, which was supported by the fact that all the hatchlings from a single nest emerged to the surface from a common path and opening (Figure 1b). Possibly, such effects could effectively reduce the collective amount of energy that the hatchlings need to expend during the digging process (Carr & Hirth, 1961; Lacroix et al., 2022; Rusli et al., 2016).

In conclusion, our suggestion that hatching is a response to diel nest temperatures indicates that shallow‐nesting *P. sinensis* has disparate nocturnal emergence strategies from those of deep‐nesting turtles (e.g., marine turtles, and possibly some freshwater turtles with larger body sizes). In general, diel variations are minor in deep nests, as the effects of solar radiation are greatly defused (Nishizawa et al., 2021; Santidrián Tomillo et al., 2017). The selection of emergence timing occurs in the process of digging to the surface (2–7 days) when marine turtle hatchlings wait at a depth that allows them to perceive a decline in temperature associated with night‐time (Glen et al., 2006; Mrosovsky, 1968; Nishizawa et al., 2021). Conversely, the nest temperature of *P. sinensis* displayed pronounced diel variations, with the onset of the drop in nest temperature broadly coinciding with the coming night (Figure 3). Because turtle embryos within the eggshell can behaviorally (Du et al., 2011; Zhao et al., 2013) and physiologically (Ye et al., 2021) respond to ambient temperatures, it is reasonable to assume that full‐term embryos could detect the arrival of night and hatch by perceiving a decline in nest temperature, further facilitating nocturnal emergence. Furthermore, nest‐temperature data available in the existing literature indicate that the eggs of other shallow‐nesting turtle species also do not experience an appreciable drop in temperature until around dusk (Doody, 2011; Witherington et al., 1990). Identifying the taxonomic and ecological diversities of thermal cues that induce the hatching of other shallow‐nesting, nocturnally emerged freshwater turtle species will lead to a better understanding of the evolutionary implications of this type of hatching strategy.

## AUTHOR CONTRIBUTIONS

**Qingjun Zhu:** Conceptualization (equal); data curation (equal); writing – original draft (equal). **Liu Lin:** Supervision (equal); writing – review and editing (equal). **Fei Kong:** Writing – review and editing (equal). **Ting Zhang:** Writing – review and editing (equal). **Hai‐Tao Shi:** Funding acquisition (equal); project administration (equal); writing – review and editing (equal).

## FUNDING INFORMATION

Financial support was provided by the National Natural Science Foundation of China (grant no. 32170532 to H.S.).

## CONFLICT OF INTEREST STATEMENT

We declare we have no competing interests.

## Supporting information


Video S1

Video S2

## Data Availability

Data are available from the Dryad Digital Repository: https://doi.org/10.5061/dryad.qbzkh18kk.
